# A data driven approach to understanding the organization of high-level visual cortex

**DOI:** 10.1038/s41598-017-03974-5

**Published:** 2017-06-15

**Authors:** David M. Watson, Timothy J. Andrews, Tom Hartley

**Affiliations:** 1 Department of Psychology and York Neuroimaging Centre, University of York, York, YO10 5DD United Kingdom; 2 School of Psychology, The University of Nottingham, Nottingham, NG7 2RD United Kingdom

## Abstract

The neural representation in scene-selective regions of human visual cortex, such as the PPA, has been linked to the semantic and categorical properties of the images. However, the extent to which patterns of neural response in these regions reflect more fundamental organizing principles is not yet clear. Existing studies generally employ stimulus conditions chosen by the experimenter, potentially obscuring the contribution of more basic stimulus dimensions. To address this issue, we used a data-driven approach to describe a large database of scenes (>100,000 images) in terms of their visual properties (orientation, spatial frequency, spatial location). K-means clustering was then used to select images from distinct regions of this feature space. Images in each cluster did not correspond to typical scene categories. Nevertheless, they elicited distinct patterns of neural response in the PPA. Moreover, the similarity of the neural response to different clusters in the PPA could be predicted by the similarity in their image properties. Interestingly, the neural response in the PPA was also predicted by perceptual responses to the scenes, but not by their semantic properties. These findings provide an image-based explanation for the emergence of higher-level representations in scene-selective regions of the human brain.

## Introduction

Human observers can reliably perceive and categorize scenes based on their spatial organisation and semantic content. These processes are thought to rely upon a network of brain regions that respond preferentially to images of scenes. These regions include the Parahippocampal Place Area (PPA)^1^, Retrosplenial Complex (RSC)^2^, and Occipital Place Area (OPA)^3^. Although studies using univariate fMRI analyses have reported comparable overall magnitudes of response to different scene categories within these regions^1^, more recent reports employing multivariate techniques have identified distinct patterns of response to different types of scene^4^, suggesting a finer-grained topographic organisation.

The organizing principles underpinning the topographic organization of scene-selective regions are the subject of current debate^5, 6^. Some studies have argued that these regions represent semantic or categorical properties of scenes^4^. For instance, models based upon assigning semantic labels to objects in scenes have been shown to predict neural responses in high-level visual cortices^5, 7–10^. However, it does not necessarily follow that patterns of neural response are systematically organised by semantic or categorical properties, or that the perception of these properties is causally linked to such patterns. Indeed, other studies have suggested that responses in these regions may be better explained in terms of the spatial characteristics of the scene such as openness^11, 12^ or distance^13^. However, it remains unclear whether such properties could themselves be explained by more basic characteristics of the scene. For example, visual properties of images can be used to classify different scene categories and to derive spatial properties such as openness^14^. Recently, we showed that such low-level visual properties predict the patterns of neural response in scene-selective regions^15, 16^, and that manipulations of visual features have a marked effect on these response patterns^17^. Taken together, these findings raise the possibility that patterns of response in scene-selective regions could be understood in terms of more basic dimensions of the stimulus.

To understand how the perception of scene categories might emerge from more basic visual characteristics of images, it is necessary to understand the way they affect patterns of response in scene selective regions. A fundamental problem in almost all univariate and multivariate studies to date is that they have relied on experimental designs which contrast responses to stimuli in different experimenter-defined categories. This makes it difficult to separate the effects of the arbitrary and subjective manipulation of category from those driven by correlated image statistics; indeed, it has been shown that measures of both visual statistics and semantic object labels significantly predict responses in scene selective cortices^5^. While many previous studies have made substantial efforts to control for such confounds, they have not necessarily overcome limitations posed by the subjective sampling of the available stimulus space.

In this study, we aim to directly compare the relative contribution of image properties and semantic features to the organization of scene-selective regions using a data-driven approach to stimulus selection in which images are chosen based only on their visual content, rather than membership of predefined categories. We used a measure of visual properties (GIST^18^) in conjunction with an unsupervised clustering algorithm to sample images from different regions of this visual feature space. The GIST model has previously been shown to be a good model of neural representations in visual cortex^15, 19^, and was designed to capture the critical visual and spatial properties thought to underlie scene perception^14, 18^, and is thus well motivated as a scene descriptor. Our aim was to determine whether the neural representation in scene-selective regions reflects fundamental visual properties. If scene-selective areas are sensitive to the visual content of scenes, we would expect to find distinct patterns of response to each cluster, and that the representational similarity of neural responses to different scene clusters would be well explained in terms of the similarity of corresponding visual descriptors.

## Results

**Figure 1:**
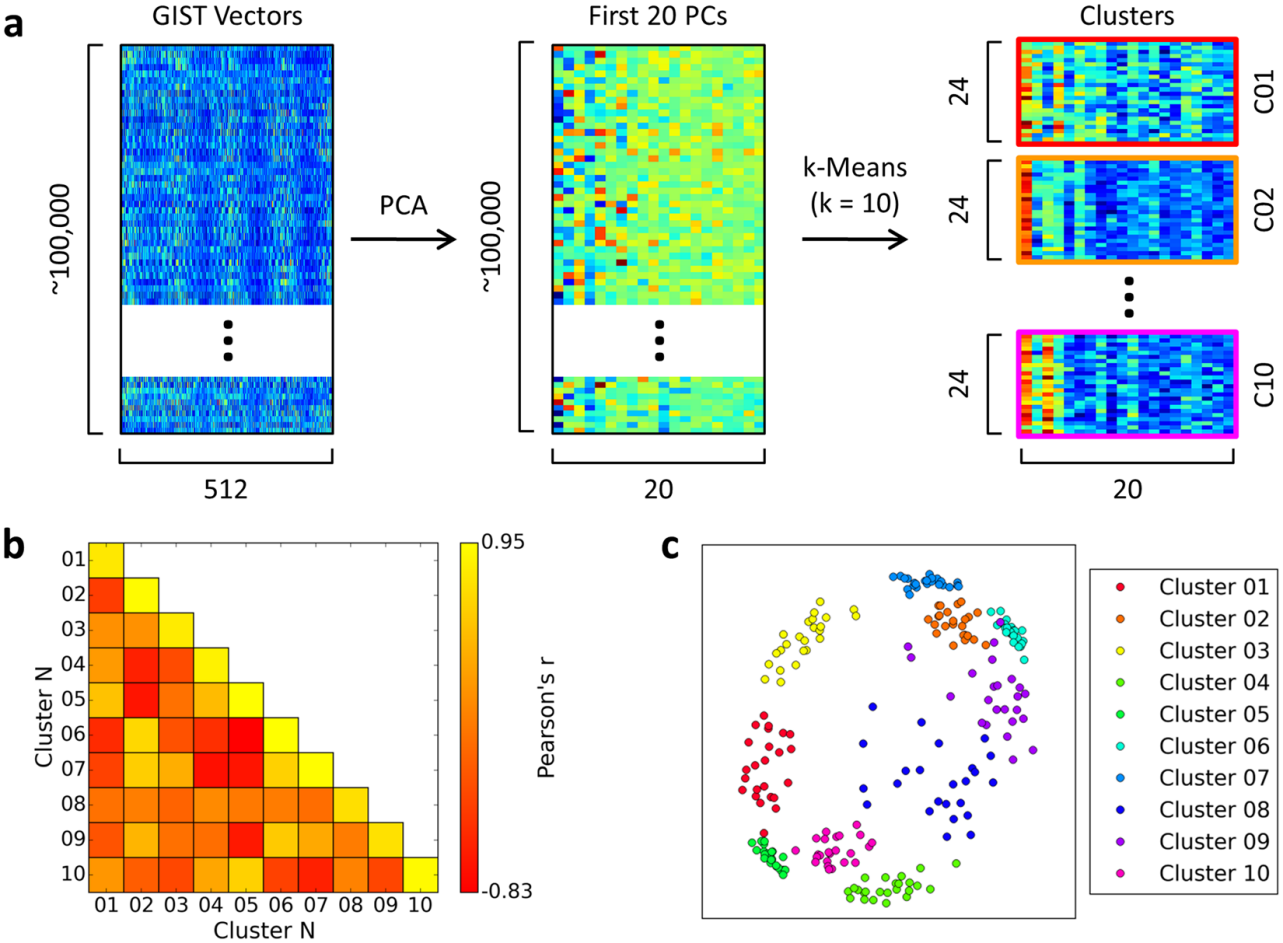
GIST clustering process. The GIST descriptor^18^ comprises a vector of 512 values that represent the image in terms of the spatial frequencies and orientations present within each of 16 spatial locations across the image. (**a**) GIST descriptor vectors were calculated for every image in the SUN database. PCA was used to reduce dimensionality down to the first 20 components, and a k-Means clustering algorithm then used to select 10 clusters of scenes. Finally, the 24 images nearest the centroid of each cluster were selected to form the final stimulus set. The structure of the feature space is illustrated by the correlations similarity matrix (**b**) and multi-dimensional scaling plots (**c**). Examples of the images from each scene cluster are available at https://figshare.com/s/a7fdfa8742abf59e3672.

A data-driven approach was used to define scene clusters based on their image properties. The GIST descriptor was applied to each of the approximately 100,000 images in the Scene Understanding (SUN) database^20^. This generates a vector of 512 values that represents each image in terms of the spatial frequencies and orientations present at different spatial locations across the image. Before applying the clustering algorithms, we first reduced the dimensionality of the GIST descriptor using principal components analysis (PCA). We then applied a k-Means clustering algorithm (k = 10) to the first 20 principal components in order to identify 10 distinct clusters of samples within this space. Finally, we selected the 24 images nearest to the centroid of each cluster. This produced a final stimulus set of 240 images, comprising 10 scene clusters each with 24 images. This process is illustrated in Fig. 1.

To help visualise the structure of the points within the feature space, we computed a correlation based similarity matrix using a leave-one-image-out cross-validation procedure. Figure 1b shows the correlations matrix averaged across the cross-validation iterations. We also used multi-dimensional scaling to provide a 2D approximation of the feature space (Fig. 1c). Example images from each scene cluster can be viewed at https://figshare.com/s/a7fdfa8742abf59e3672. In addition, the entire stimulus set is viewable at https://figshare.com/s/71a735b27bcf0db53360. It is clear that these images do not reflect the scene categories commonly used in previous studies. For instance, cluster 6 is marked by images with a strong horizontal component across the middle of the image – this frequently manifests as a horizon line, though there are also other instances that conform to the rule (including some indoor scenes). Importantly, images within this cluster span a range of semantic categories, including indoor and outdoor scenes, and manmade and natural scenes.

**Figure 2:**
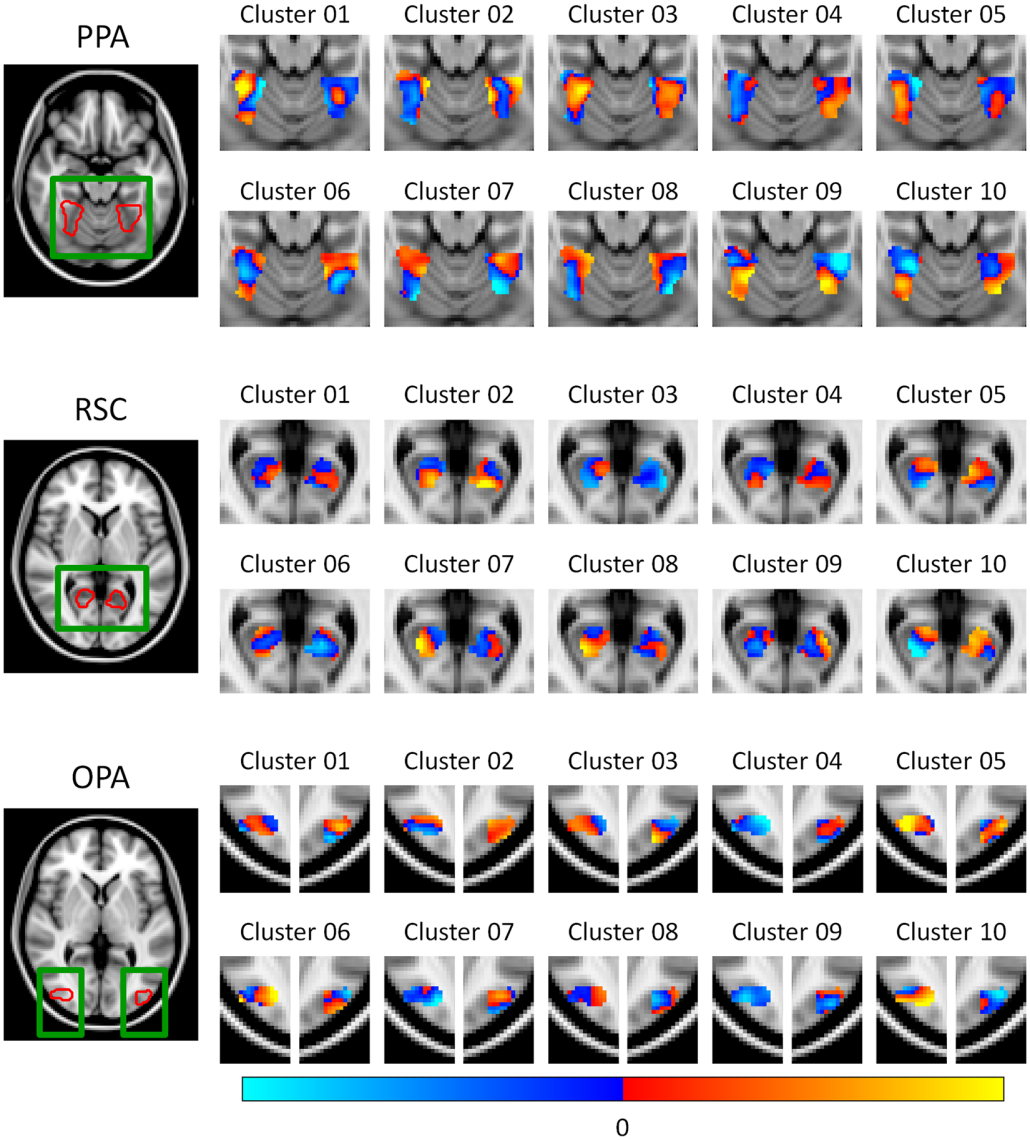
Group patterns of response restricted to each of the scene-selective regions (PPA, RSC, OPA). Responses are normalized by subtracting a voxel-wise mean across all conditions, such that red and blue colours indicate values above and below the mean response respectively.

An independent localiser scan was used to identify regions of interest (ROIs) for the 3 core scene-selective regions (PPA, RSC, and OPA) by comparing the responses to intact and phase scrambled images of scenes. The locations of these regions are illustrated in Supplementary Figure S1, and MNI co-ordinates of the peak voxels are given in Table 1. We measured the pattern of neural response in each scene region to the 10 different scene clusters using a blocked fMRI design. Figure 2 shows the normalised responses within each of the scene-selective regions; red and blue colours indicate responses above and below the voxel-wise mean respectively.

**Table 1:** Peak MNI mm co-ordinates, voxel counts, and thresholds of standard scene-selective clusters (PPA, RSC, OPA).

Region	Hemisphere	*x*	*y*	*z*	Voxel count	Threshold (Z)
PPA	L	−34	−46	−22	500	5.06
	R	26	−50	−18	500	5.59
RSC	L	−18	−52	−2	500	4.63
	R	16	−58	6	502	4.79
OPA	L	−36	−90	2	500	5.14
	R	38	−82	4	499	5.03

**Figure 3:**
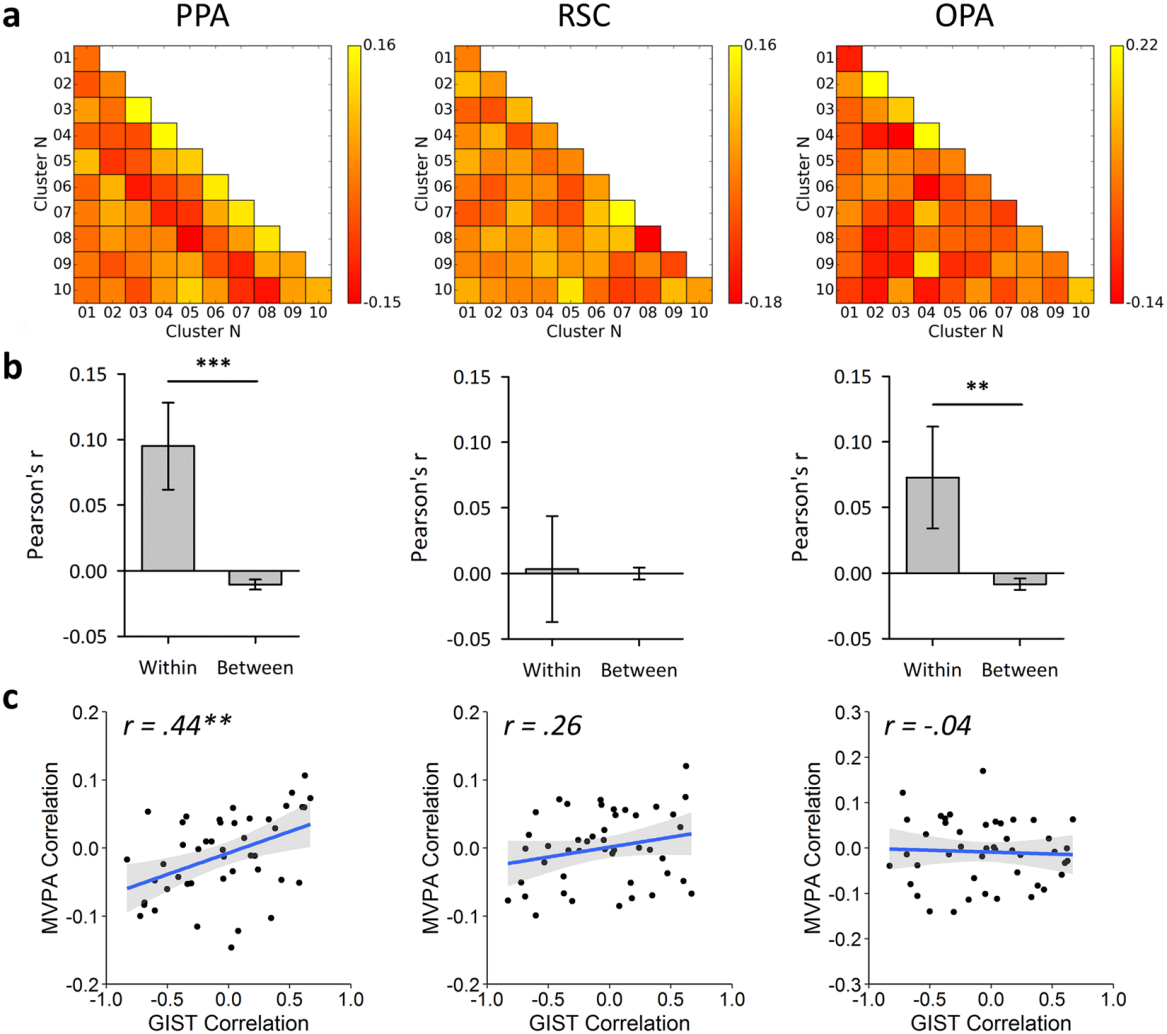
Main fMRI analyses for each scene region. (**a**) MVPA correlation similarity matrices. (**b**) Discrimination of scene clusters by contrasting within over between cluster correlation values; error bars represent 95% confidence intervals. (**c**) Results of representational similarity analyses between the off-diagonal elements of the MVPA and GIST model similarity matrices; shaded regions represent 95% confidence intervals. (***p < 0.001, **p < 0.01, *p < 0.05).

Correlation-based MVPA^21^ was used to assess the reliability of these responses. Average correlation similarity matrices for each of the scene regions are shown in Fig. 3a. A Fisher’s Z-transform was applied to the correlation values prior to further analysis, and in all cases a Bonferroni-Holm correction for multiple comparisons was applied across the 3 ROIs (PPA, RSC, OPA). We first assessed the ability of the MVPA to discriminate the scene clusters by comparing the within-cluster (on-diagonal) and between-cluster (off-diagonal) values of the correlation matrices. Figure 3b shows that there were significantly greater within- than between-cluster correlations in the PPA (t(19) = 5.98, *p* < 0.001, Cohen’s d = 1.34) and OPA (t(19) = 3.98, *p* = 0.002, Cohen’s d = 0.89), but not in the RSC (t(19) = 0.11, *p* *=* 0.918, Cohen’s d = 0.02). These effect sizes remained relatively stable across a range of sizes of the ROI definitions (Supplementary Figure S2a). This shows that there are distinct neural responses in both PPA and OPA to the scene clusters defined by the data-driven method. However, the absence of distinct patterns in the RSC shows that this result is not an inevitable consequence of the data-driven approach to image selection.

Although these results suggest that the PPA and OPA are sensitive to the visual properties differing between the scene clusters, this does not guarantee that they are representing these properties in the same way as predicted by the GIST model. For instance, these regions could be sensitive to specific subsets or conjunctions of visual features characterised by the GIST. Nevertheless, if neural responses are organised according to such features we would expect the similarity in patterns of neural response to different scene clusters to be predicted by the similarity in the low-level image properties as defined by the GIST descriptor. To test this idea we used a representational similarity analysis^22^ to compare off-diagonal elements of the correlation matrices for each region (Fig. 3a) with the GIST correlations matrix (Fig. 1b). Results of these analyses are illustrated in Fig. 3c and show that the image properties significantly correlated with neural responses in the PPA (*r*(43) = 0.44, *p* = 0.008), but not the RSC (*r*(43) = 0.26, *p* = 0.182) or OPA (*r*(43) = −0.04, *p* = 0.770). These effect sizes remained relatively stable across a range of sizes of the ROI definitions (Supplementary Figure S2b). Again, the absence of significant representational similarity with the GIST in the OPA and RSC shows that the correlation between neural responses and image properties in the PPA is not an inevitable consequence of the data-driven approach to image selection.

**Figure 4:**
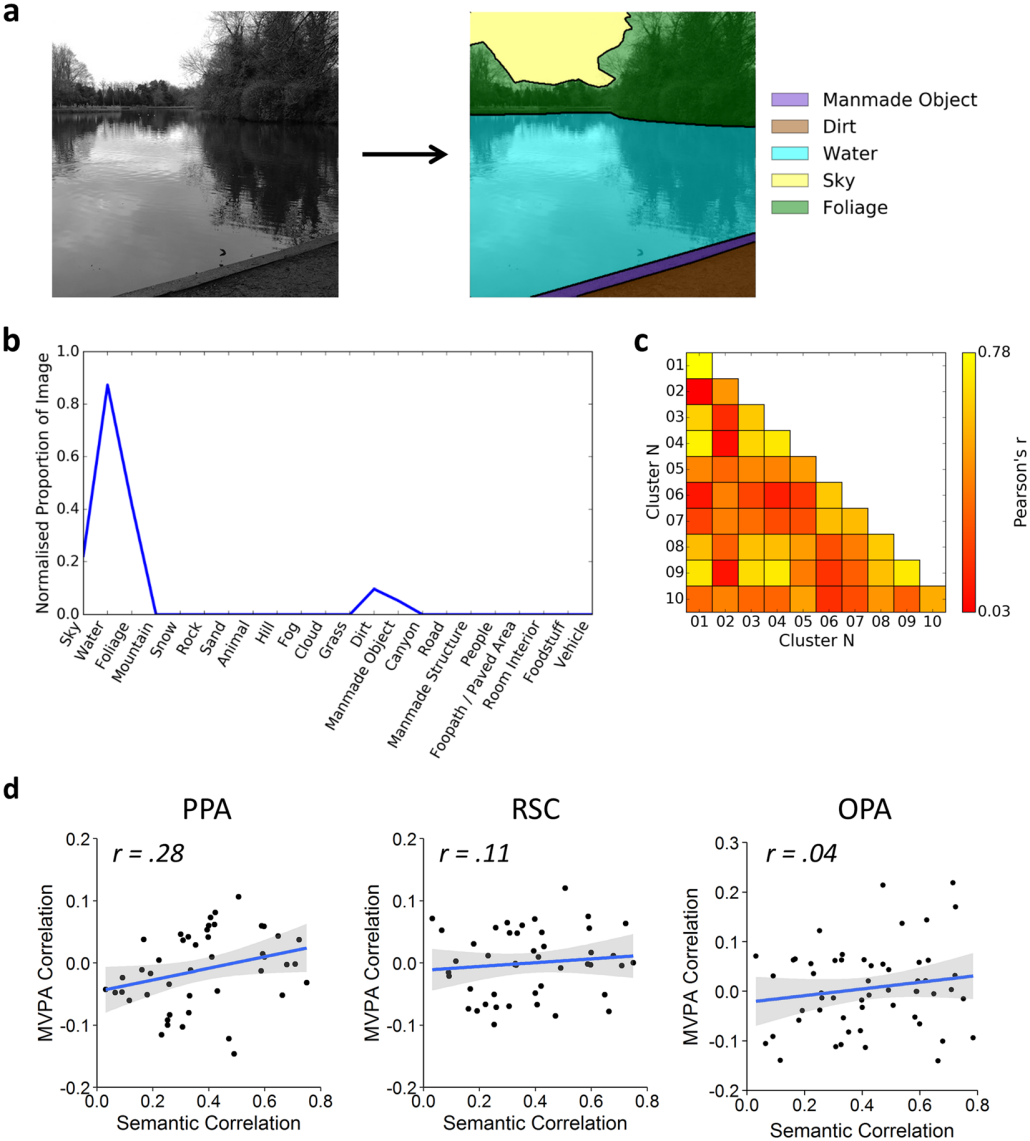
Local semantic concept model^23^. (**a**) Objects within each of the images in the stimulus set were segmented and labelled using the LabelMe toolbox^53^. Object labels were then reduced to a core set of 22 labels sufficient to describe the stimulus set. For copyright reasons, the scene image included here is an example only and is not part of the stimulus set. (**b**) For each image, a vector was constructed representing the proportion of pixels in the image occupied by each of the object labels. Vectors were normalised to have an overall magnitude of 1. (**c**) Group average similarity matrix calculated by correlating the vectors within and between clusters using a leave-one-image-out cross-validation scheme. (**d**) Results of representational similarity analyses between the off-diagonal elements of the MVPA and local semantic concept model similarity matrices; shaded regions represent 95% confidence intervals. (***p < 0.001, **p < 0.01, *p < 0.05).

Although images in each cluster were selected solely on the basis of their visual properties, it remains possible that semantic information could reliably discriminate between the clusters. For instance, scenes containing semantically similar objects may also tend to be visually similar. To address this issue, a local semantic concept model^23^ was used to test the semantic similarity of images. This defines vectors representing the objects present in each scene (Fig. 4a,b), and a similarity matrix was then produced by correlating these vectors within and between the clusters using a leave-one-image-out cross-validation procedure (Fig. 4c). To determine if each image cluster conveys distinct semantic information, we compared the within-cluster (on-diagonal) and between-cluster (off-diagonal) values of the correlation matrix. A paired-samples t-test revealed significantly higher within- than between-cluster correlations (t(23) = 12.67, *p* < 0.001, Cohen’s d = 2.59), indicating that clusters could be discriminated based on semantic properties. We next determined the representational similarity between off-diagonal elements of the local semantic properties and the image properties given by the GIST analysis. We found a positive correlation between semantic and image properties that was borderline significant (*r*(43) = 0.29, *p* = 0.050).

We next asked whether the local semantic properties could predict the patterns of fMRI response in scene-selective regions by correlating off-diagonal elements of the respective correlation matrices. Results of these analyses are illustrated in Fig. 4d. Semantic properties did not significantly correlate with neural responses for any region (PPA: *r*(43) = 0.28, *p* = 0.193; RSC: *r*(43) = 0.11, *p* = 0.942; OPA: *r*(43) = 0.04, *p* = 0.942). These effect sizes remained relatively stable across a range of sizes of the ROI definitions (Supplementary Figure S2c).

**Figure 5:**
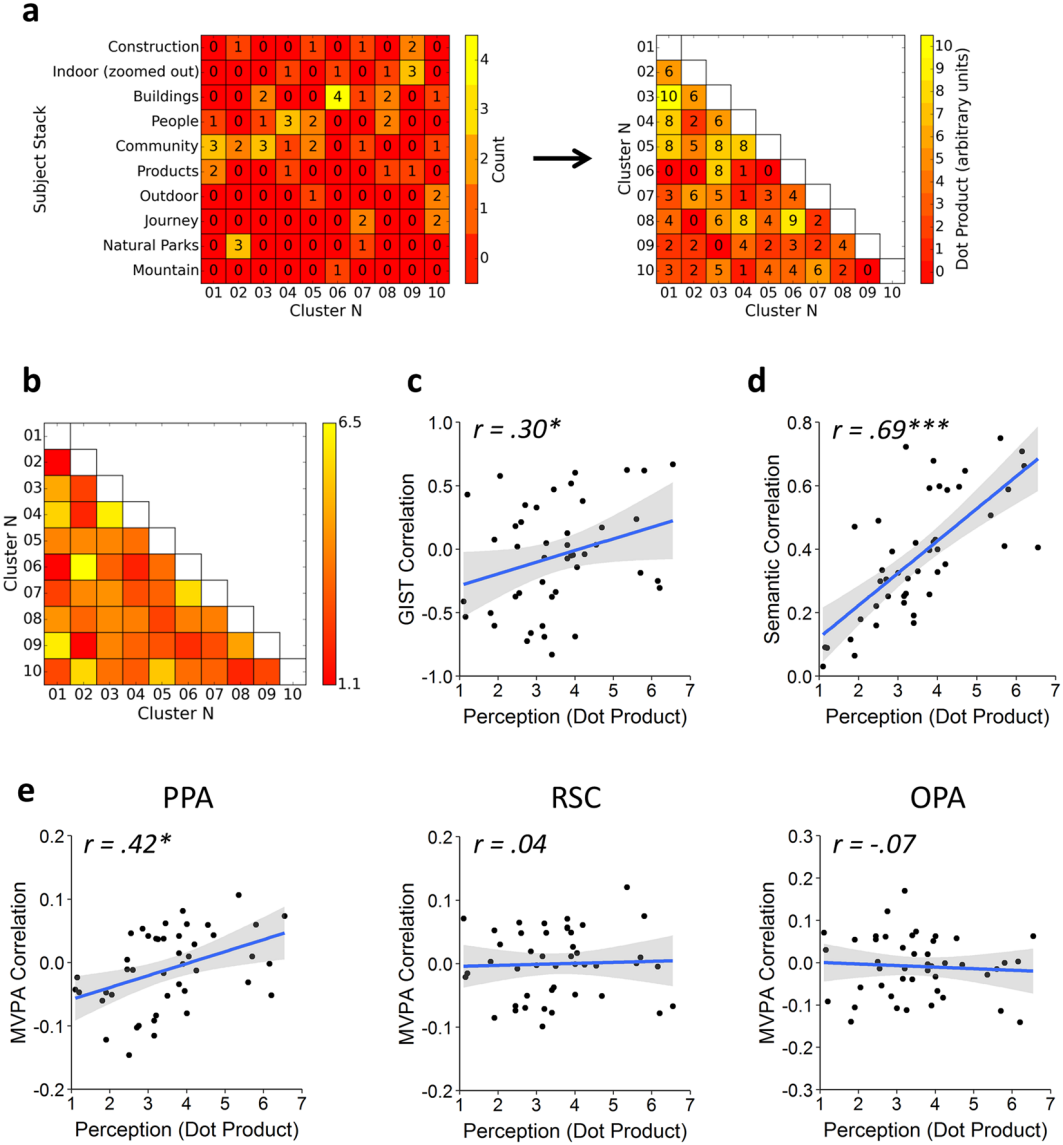
Behavioural experiment method and results. (**a**) Illustration of the analysis procedure for an example subject. A matrix of counts (left) was generated for each of the scene clusters (columns) against each of the subject’s card stacks (rows). The card stack labels were generated by the subject themselves. The lower triangle of a “perceptual” similarity matrix (right) was then constructed by calculating the dot-product between each pairwise combination of columns in the counts matrix. The group average dot product similarity matrix (**b**) was then compared against the off-diagonal elements of the: (**c**) GIST model, (**d**) local semantic concept model, and (**e**) MVPA similarity matrices in a series of representational similarity analyses. Shaded regions on scatterplots indicate 95% confidence intervals. (***p < 0.001, **p < 0.01, *p < 0.05).

To determine how the GIST and semantic models predicted human perception, participants completed a card-sorting task in which cards depicting the scenes were sorted into distinct stacks according to their perceptual similarity^24^. A similarity matrix was constructed by examining the co-occurrence of each possible pairing of scene clusters across each of the subject’s card stacks. This was calculated by defining a vector for each scene cluster denoting the counts across each of the card stacks, and then taking the dot product between each pairwise combination of vectors (Fig. 5a). The average dot product similarity matrix across subjects is shown in Fig. 5b. We first tested the representational similarity with the GIST and semantic models; results of these analyses are shown in Fig. 5c,d. A significant correlation was found between the perceptual responses and both the GIST (*r*(43) = 0.30, *p* = 0.045) and semantic models (*r*(43) = 0.69, *p* < 0.001). However, because the semantic and GIST models are themselves correlated, it remains unclear whether each model is able to explain significantly more variance in the perceptual responses beyond that already explained by the other model. To this end, we repeated our analyses using partial correlations to control for the effects of one or the other model. Results revealed a significant partial correlation between the perceptual and semantic models while controlling for the GIST model (*r*(42) = 0.66, *p* < 0.001). However, the partial correlation between the perceptual and GIST models while controlling for the semantic model failed to reach significance (*r*(42) = 0.14, *p* = 0.361). An alternative approach is to compute the semi-partial correlation such that variance between the predictor variables themselves is held constant but variance between the outcome and the second predictor variable remains unfiltered – this approach is more appropriate for directly comparing two competing predictor variables; these results are shown in Supplementary Table S2, and are consistent with those of the partial correlations. This shows that, in contrast to the neural responses, the perceptual responses were primarily predicted by the semantic model rather than the GIST model.

We next asked whether the perceptual responses could predict patterns of neural response (Fig. 5e). Perceptual responses significantly correlated with neural responses in the PPA (*r*(43) *=* 0.42, *p* = 0.012), but not the RSC (*r*(43) = 0.04, *p* > 0.999) or OPA (*r*(43) = −0.07, *p* > 0.999). These effect sizes remained relatively stable across a range of sizes of the ROI definitions (Supplementary Figure S2d). In order to compare the unique contributions of the perceptual and visual models to the PPA response, we repeated our analyses using partial correlations. Significant correlations were observed both when comparing the neural response with the GIST while controlling for the perceptual model (*r*(42) = 0.36, *p* = 0.017), and comparing the neural response with the perceptual model while controlling for the GIST (*r*(42) = 0.34, *p* = 0.024). Repeating these analyses as semi-partial correlations revealed a similar pattern of results (Supplementary Table S2). Thus, the visual and perceptual models accounted for distinct components of the variance in the PPA response.

Our results show that patterns of neural responses in the PPA are linked to the image properties of scenes. However, previous studies have suggested possible divisions of function between the posterior and anterior aspects of the PPA^25, 26^. To test this possibility, we repeated our analyses within posterior and anterior sub-divisions of our PPA region. We found distinct patterns of response to clusters of scenes defined by our data-driven approach in both the anterior and posterior sub-divisions. We also found that the GIST and perceptual (but not semantic) models significantly predicted neural responses in the anterior PPA. By contrast, none of the models significantly predicted responses in the posterior PPA. These results are illustrated in Supplementary Figure S3.

In the PPA, the similarity of the neural patterns of response to different clusters could be predicted by the similarity in their image properties. However, a corresponding effect is not evident in the OPA or RSC. To determine whether this might reflect overall differences in the neural response across these regions, we tested the reliability of both univariate and multivariate responses. Results of these analyses are shown in Supplementary Figure S4. We first calculated the mean univariate amplitude of response in each region for each of the scene clusters (Supplementary Figure S4a). Responses were significantly greater than zero for all scene clusters in PPA and OPA, and for 7 out of 10 clusters in RSC. However, response amplitudes were relatively greater in the PPA and OPA than in the RSC. We next tested the reliability of the MVPA correlation matrices (Supplementary Figure S4b). For each region, a noise ceiling was calculated, which estimates the maximum representational similarity that is achievable given the noise in the data^27^. Higher noise ceilings indicate more reliable neural responses. There was substantial overlap in the noise ceiling across the different scene-selective regions. This demonstrates a similar reliability in the patterns of response in each region. We additionally tested the variability in the representational similarity between the MVPA and each of the models (GIST, semantic, and perceptual) by correlating each model with the MVPA matrix for each LOPO iteration in turn. Results mirrored those of the main RSAs; GIST and perceptual models significantly predicted neural responses in the PPA, whilst no other comparisons were significant.

**Figure 6:**
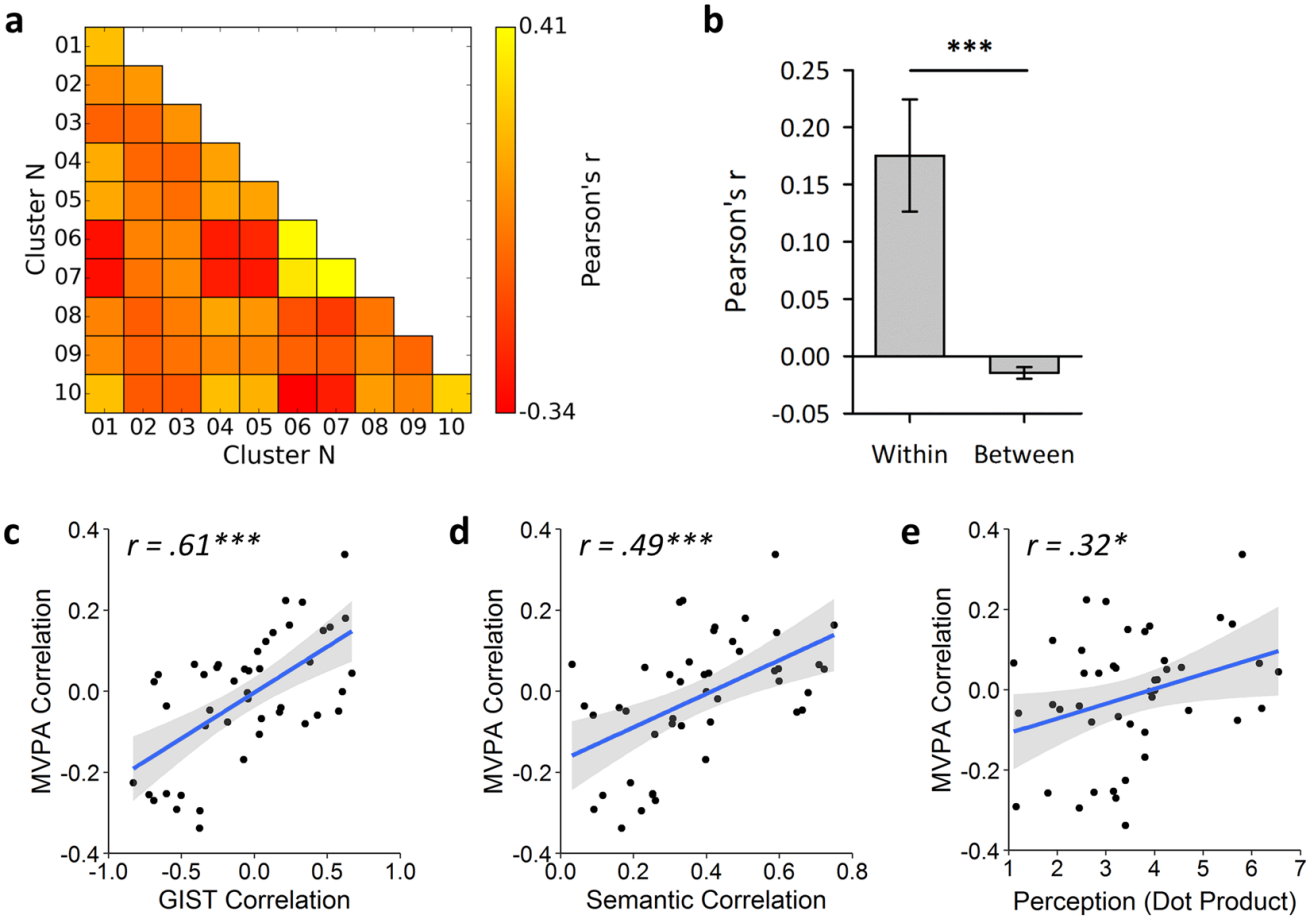
Results from analysis of V1 control region. (**a**) MVPA correlations matrix. (**b**) Discrimination of scene clusters by contrasting within over between cluster correlation values; error bars represent 95% confidence intervals. Also shown are the results of representational similarity analyses between the off-diagonal elements of the MVPA and: (**c**) GIST, (**d**) semantic, and (**e**) perceptual models; shaded regions indicate 95% confidence intervals. (***p < 0.001, **p < 0.01, *p < 0.05).

If visual properties serve to organize patterns of neural response in scene selective areas we might expect to see a similar pattern of results in early visual areas, where they are known to be systematically organized within retinotopic maps. To investigate this, we repeated our analyses in a V1 control region. Results of these analyses are shown in Fig. 6. Firstly, significantly higher within- than between-cluster correlations were observed (t(19) = 7.34, *p* < 0.001, Cohen’s d = 1.64), indicating that patterns of response could be discriminated. Next, a representational similarity analysis revealed a significant correlation between neural responses and image properties (*r*(43) = 0.61, *p* < 0.001). A more modest, but nevertheless statistically significant correlation was also observed between neural responses and local semantic properties (*r*(43) = 0.49, *p* < 0.001). Repeating these analyses as partial correlations revealed significant correlations between neural responses and the GIST model while controlling for the semantic model (*r*(42) = 0.56, *p* < 0.001), and between neural responses and the semantic model while controlling for the GIST model (*r*(42) = 0.41, *p* *=* 0.005). Similar results were obtained repeating these analyses as semi-partial correlations (Supplementary Table S2). Finally, a significant correlation was observed between neural and perceptual responses (*r*(43) = 0.32, *p* = 0.031). Using partial correlations, a significant correlation was observed between the neural response and the GIST while controlling for the perceptual model (*r*(42) = 0.57, *p* < 0.001). However, the correlation between the neural response and the perceptual model while controlling for the GIST failed to reach significance (*r*(42) = 0.18, *p* = 0.234). Repeating these analyses as semi-partial correlations yielded similar results (Supplementary Table S2). Thus, the GIST model proved a better predictor of the V1 response than the perceptual model.

**Figure 7:**
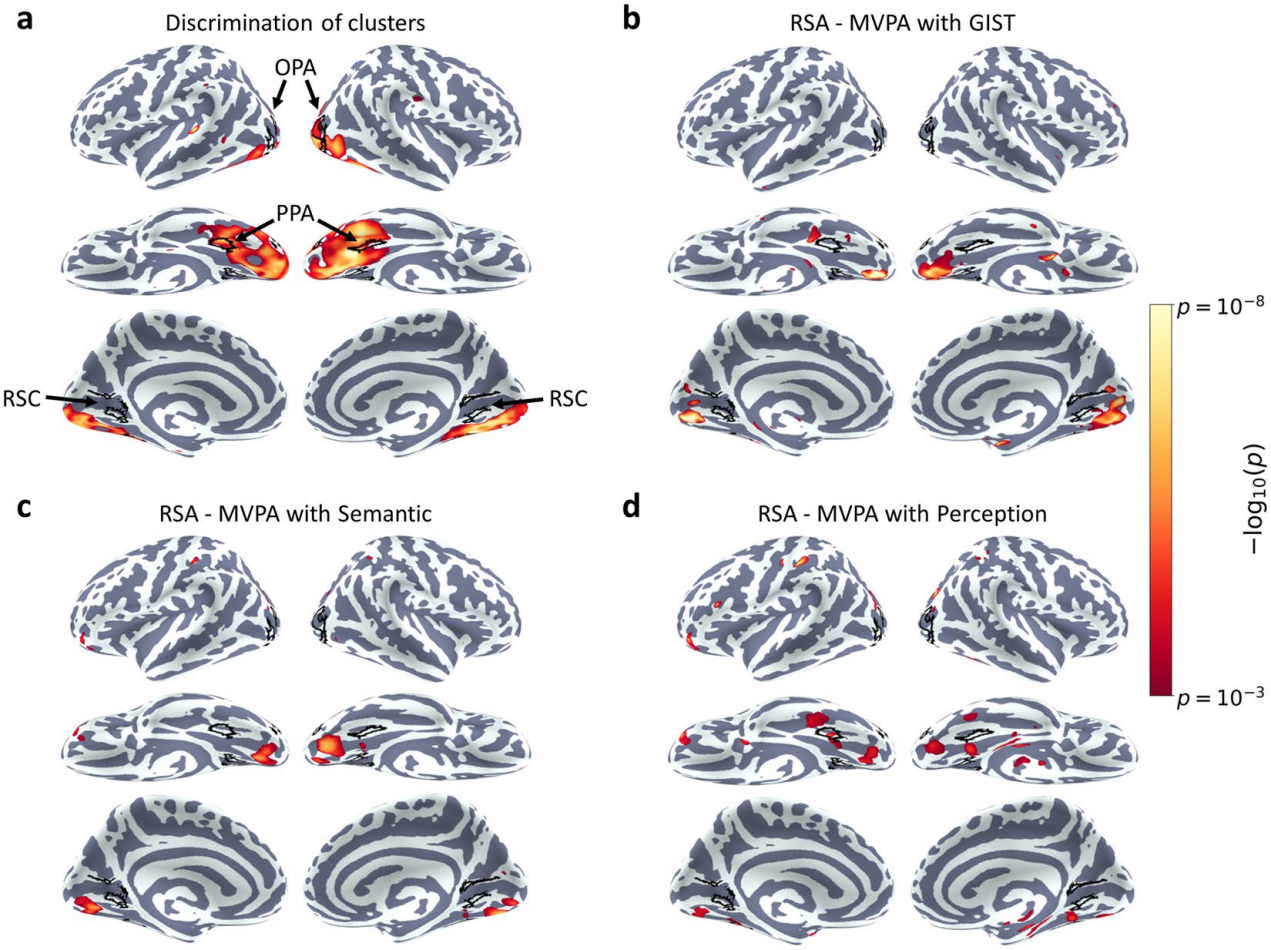
Searchlight analyses. (**a**) Discrimination of scene clusters, assessed by paired-samples t-tests contrasting within over between cluster correlations. (**b**–**d**) Results of representational similarity analyses between off-diagonal elements of the MVPA and (**b**) GIST, (**c**) semantic, and (**d**) perceptual models. In all cases the relevant test was run for each sphere separately, and the resulting *p*-value assigned to the central voxel of the sphere. These *p*-statistic maps are displayed on the Freesurfer average surface, thresholded at *p* < 0.001 (uncorrected). Locations of the main scene ROIs are also highlighted.

Finally, to test for effects outside of our main ROIs, we repeated our analyses using a searchlight approach^28^. Figure 7a shows the results of paired-samples t-tests of the within- over between-cluster correlation values for each sphere mapped to the cortical surface. Significant discrimination of scene clusters was observed throughout occipital and ventro-temporal visual cortices. We also performed the simple representational similarity analyses using the searchlight approach, comparing the MVPA similarity matrices against the GIST, local semantic concept, and perceptual models. For all three models, results primarily highlighted significant spheres in early visual and posterior ventro-temporal visual cortices, although some more anterior clusters were also observed (Fig. 7b–d). However, it is important to note that the searchlight approach is limited to regions that have small spherical representations^29^, and that this may explain why more significant clusters were not found in more anterior regions of the temporal lobe.

## Discussion

The aim of this study was to explore the functional organization of scene-selective regions in the human brain using a data-driven approach. Clusters of scenes were defined objectively by their image properties. Importantly, these clusters did not correspond to commonly defined scene categories. Nevertheless, we found distinct patterns of response to the scene clusters in the scene-selective PPA and OPA regions. The similarity of neural responses in the PPA to different scene clusters was well explained by the similarity of the corresponding visual descriptors. These results suggest that the established higher-level representational structure in PPA emerges from a more basic organizational framework reflecting the visual properties of the image.

Previous studies have revealed distinct patterns of neural response within scene-selective regions to scene categories^4^, and spatial properties such as openness^11, 12^ or distance^13^. Our findings suggest that the topographic organization of the PPA is sensitive to the visual properties of the image. We found that visually defined clusters generated distinct patterns of response in the PPA, and these responses showed a similar representational structure to that predicted by the GIST model^18^. At first sight, this analysis may appear somewhat circular, in the sense that the GIST model is used to predict responses to scene clusters initially defined by the same model. However, we would not expect responses to track visual similarity so closely in regions where representations are organized according to unrelated characteristics. Indeed, our findings from other scene-selective regions show that the patterns of response in the PPA are not an inevitable consequence of the data driven approach. For example, we did not observe any significant correlation between GIST and the representational similarity structure in the OPA or RSC. So rather than being a consequence of our approach, we believe this shows that the organisation of responses in PPA reflects visual properties similar to those captured by the GIST model. In this regard, the findings of this study are consistent with previous studies that have demonstrated associations between GIST and neural representations for different semantic categories of scenes^15^ and objects^19^.

Because the scene clusters used in the current study are essentially unrelated to the scene categories used in classic designs, our results demonstrate that scene category need not be considered the dominant organising principle of scene-selective regions. Rather, category-level responses are likely driven by systematic variation in more basic properties that are associated with particular scene categories. These findings fit with previous studies showing that visual properties of scenes can predict responses in scene-selective regions^15^. Similar conclusions are evident in studies that have reported sensitivity in scene-selective regions for orientation^30, 31^, spatial frequency^32^, and visual field location^33, 34^. Together, these findings suggest an important role of more basic visual dimensions of the stimulus, perhaps extending organizing principles that govern the functional topography of early visual cortex^35^.

The theoretical implications of the relationship between low- and high-level properties are more nuanced, however. We are not arguing that the PPA is merely involved in low-level visual processing and our results do not “explain away” earlier findings concerning semantic and spatial correlates. The GIST model has been shown to provide a computational basis for scene categorization and is related to spatial parameters such as openness and distance^14^. This suggests a mechanism through which specific image features can be analysed to guide spatial behaviour, thus linking low- and high-level vision. Consistent with this perspective we found that images from each cluster had distinct semantic properties. Moreover, similarity in visual properties between clusters was weakly correlated with the similarity in their local semantic features, and we observed a non-significant but non-negligible association between patterns of neural response and semantic properties. In this context, it seems likely that the distinct patterns of response in the PPA associated with varying spatial parameters and categorical distinctions are related to the function of mediating spatial behaviour (i.e. they emphasise those visual parameters that distinguish different semantic or spatial properties). This suggests a bottom-up mechanism for the emergence of higher-level representations in the scene-selective cortex. More anterior regions such as the anterior aspects of parahippocampal gyrus, the entorhinal cortex, and the hippocampus have been implicated in the processing of additional higher level aspects of scenes, such as route learning and spatial navigation^36, 37^. Further behaviourally relevant aspects of scene processing may therefore be supported by such regions, potentially mediated by their connectivity with more posterior scene-selective regions in ventral visual cortex^25, 38, 39^.

There are many other statistical descriptors of images available besides the GIST model. Had an alternative model been used with the data driven approach it is possible that a different scene cluster structure may have been obtained, and it is possible that an alternative model may prove a better predictor of neural responses. Nevertheless, the use of the GIST model is well justified for a number of reasons. Firstly, the GIST was explicitly designed to capture the critical visual-spatial features of scenes thought to underlie human scene perception^14, 18^, whilst many other models are not specifically motivated for scenes. Secondly, the GIST model has previously been shown to provide a good model of neural representations in ventral visual cortices^15, 19^. The GIST descriptor therefore provides a reasonable approximation of how visual information may be represented in scene-selective cortices. Nevertheless, future research may further investigate the relationship between alternative models and representations in high level visual cortices. For example, Ҫukur and colleagues found that the addition of motion to a low-level model was able to explain additional variance in the neural response to movies of scenes^9^. Other studies have suggested that deep convolutional neural networks may offer a richer source of information about the underlying neural representations than simpler models^40–42^.

How do neural patterns of response contribute to the perception of scenes? Many previous studies have demonstrated our ability to categorise scenes^20, 43, 44^. However, like the earlier neuroimaging research, these studies typically rely on tasks that are constrained by the choice of categories. Here, we used a card-sorting task that allowed participants a high degree of freedom in choosing how to group scenes^24^. Both visual and perceptual models were correlated with the representational similarity of neural responses in the PPA, and each explained relatively independent components of the variance. Such findings are consistent with previous reports showing a relationship between PPA function and human behaviour^4^. Yet, while the visual model provided the better account of patterns of neural response, the semantic model explained the most variance in participants’ perceptual responses, suggesting a partial dissociation between the mechanisms driving patterns of neural response in PPA and those responsible for perceptual judgements.

The results from the PPA demonstrate that our methods are sensitive to visual organization, but this is not seen throughout scene-selective cortex. Our data-driven approach only leads to a correspondence between neural representation and visual characteristics in regions where the underlying organization is coupled to image properties, and our findings suggest that RSC and, to some extent, the OPA are not organized in this way. RSC responses failed to discriminate the scene clusters, and the representational similarity structure was not predicted by any of the models tested. Many previous studies have identified complimentary but distinct roles for the PPA and RSC, with the PPA proposed to be processing spatial features in the immediate visual environment while the RSC focuses on integrating the scene within the wider spatial environment^26, 45^. A somewhat different pattern of results was observed in the OPA. Although distinct patterns of response to different scene clusters were evident in the OPA, the representational similarity of the neural responses was not predicted by GIST. Furthermore, neither the semantic nor the perceptual models predicted the representational similarity structure seen in the OPA.

In conclusion, we describe a method for analysing neural responses to scenes using data-driven clustering to select stimuli based on objective image properties. This overcomes limitations of classic experimental designs in which stimuli are subjectively allocated to predefined categories. We found that these image clusters elicited distinct patterns of response in the PPA. In addition, the similarity in the patterns of response to different clusters could be predicted by the similarity in their image properties. Finally, the neural response in the PPA was also predicted by perceptual responses to scenes, but not by their semantic properties. Overall, the results underscore the importance of visual features in the emergence of higher-level representations in the PPA.

## Methods

### Participants

20 participants (5 males, 15 females; mean age: 25.8; age range: 19–34) took part in the experiment. All participants were neurologically healthy, right-handed, and had normal or corrected-to-normal vision. The study was approved by the York Neuroimaging Centre Ethics Committee, and all methods were performed in accordance with the relevant guidelines and regulations of the committee. Informed written consent for study participation was obtained from all participants.

### Data-Driven Image Selection

The experimental stimulus set was generated by an entirely data-driven approach. In order to reflect the high variability of real world scenes we selected images from the SUN397 database^20^ as this offers a large (over 100,000 images) and diverse range of scenes. Image properties were measured with the GIST descriptor^18^. The GIST descriptor uses a vector of 512 values to represent an image in terms of the spatial frequencies and orientations present at different spatial locations across the image.

Images were first cropped and resized to the resolution that they would be presented at in the experiment (256 × 256 pixels) and converted to grayscale. A GIST descriptor was then calculated for every image in the SUN database. GIST vectors were normalised by first scaling each component of the vectors to sum to 1 across images, and second by scaling each vector to have a magnitude of 1. Each image is thus represented as a point in a 512-dimensional feature space by its normalised GIST descriptor. Attempting to apply clustering algorithms in such a high-dimensional space can be problematic, so we first reduced the dimensionality using principal components analysis (PCA). The first 20 principal components were selected; these explained 70.35% of the variance of the original components. We applied a k-Means clustering algorithm (k = 10; Euclidean distance metric) to identify 10 distinct clusters of samples within this space, such that samples within a cluster are defined by having similar image properties to one another. This number of clusters was selected as it represented a feasible number of conditions to fit within the time constraints of an MRI scan run. Finally, we selected the 24 points nearest the centroid of each cluster as measured by Euclidean distance. The GIST descriptor is not sensitive to colour, so images were presented in greyscale. Mean luminance and visual RMS contrast were equated across images. Example images from each scene cluster can be viewed at https://figshare.com/s/a7fdfa8742abf59e3672. In addition, the entire stimulus set is viewable at https://figshare.com/s/71a735b27bcf0db53360.

PCA and k-Means algorithms were implemented using the Python Scikit-learn toolbox^46^. A correlations similarity matrix was constructed by correlating the principal component vectors within and between scene clusters using a leave-one-image-out cross-validation procedure. For each cluster, the principal component vectors were averaged across all but one of the images, and the average and left-out vectors correlated within and between clusters. This process was then repeated so that every image was left out once. The structure of the selected scene space was also visualised in two dimensions using multi-dimensional scaling.

### fMRI Experimental Design

Visual stimuli were back-projected onto a custom in-bore acrylic screen at a distance of approximately 57 cm from the participant, with all images presented at a resolution of 256 × 256 pixels subtending approximately 10.7 degrees of visual angle. Images presented in both the experiment and localiser runs were taken from the SUN database^20^ (http://groups.csail.mit.edu/vision/SUN/). Stimuli were presented using PsychoPy^47, 48^.

During the experimental scan, participants viewed images from the 10 scene clusters. Images from each condition were presented in a blocked fMRI design, with each block comprising 6 images. Each image was presented for 750ms followed by a 250ms grey screen that was equal in mean luminance to the scene images. Each stimulus block was separated by a 9 s period in which the same grey screen as used in the inter-stimulus interval was presented. Each condition was repeated 4 times giving a total of 40 blocks. To maintain attention throughout the scan participants performed a passive task detecting the presence of a red dot randomly superimposed on one of the images in each block, responding via a button press.

An independent localiser scan was used to define scene-selective regions. During the localiser scan, participants viewed images from 2 stimulus conditions: (1) intact scene images and (2) phase scrambled versions of the same images in condition 1. Images from each condition were presented in a blocked fMRI design, with each block comprising 9 images. Each block was separated by a 9 s period in which the same grey screen was presented. Each condition was repeated 8 times giving a total of 16 blocks. To maintain attention participants performed a one-back task detecting the presentation of a repeated image in each block, responding via a button press.

### Imaging Parameters

All scanning was conducted at the York Neuroimaging Centre (YNiC) using a GE 3 Tesla HDx Excite MRI scanner. Images were acquired with an 8-channel phased-array head coil tuned to 127.72 MHz. Data were collected from 38 contigual axial slices in an interleaved order via a gradient-echo EPI sequence (TR = 3 s, TE = 32.5ms, FOV = 288 × 288 mm, matrix size = 128 × 128, voxel dimensions = 2.25 × 2.25 mm, slice thickness = 3 mm with no inter-slice gap, flip angle = 90°, phase-encoding direction = anterior-posterior, pixel bandwidth = 39.06 kHz). In order to aid co-registration to structural images, T1-weighted in-plane FLAIR images were acquired (TR = 2.5 s, TE = 9.98ms, FOV = 288 × 288 mm, matrix size = 512 × 512, voxel dimensions = 0.56 × 0.56 mm, slice thickness = 3 mm, flip angle = 90°). Finally, high-resolution T1-weighted structural images were acquired (TR = 7.96ms, TE = 3.05ms, FOV = 290 × 290 mm, matrix size = 256 × 256, voxel dimensions = 1.13 × 1.13 mm, slice thickness = 1 mm, flip angle = 20°).

### fMRI Analysis

Univariate analyses of the fMRI data were performed with FEAT v5.98 (http://www.fmrib.ox.ac.uk/fsl). In all scans the initial 9 s of data were removed to reduce the effects of magnetic stimulation. Motion correction (MCFLIRT, FSL^49^) was applied followed by temporal high-pass filtering (Gaussian-weighted least-squared straight line fittings, sigma = 15 s). Spatial smoothing (Gaussian) was applied at 6 mm FWHM to both the localiser and experiment runs, in line with previous studies employing smoothing in conjunction with MVPA^15, 50^. Parameter estimates were generated for each condition by regressing the hemodynamic response of each voxel against a box-car regressor convolved with a single-gamma HRF. Head motion parameters were also included as confound regressors. Next, individual participant data were entered into higher-level group analyses using a mixed-effects design (FLAME, FSL). Functional data were first co-registered to an in-plane FLAIR anatomical image then to a high-resolution T1-anatomical image, and finally onto the standard MNI brain (ICBM152).

An independent localiser scan was used to define regions of interest (ROIs) for the 3 main scene-selective regions: Parahippocampal Place Area (PPA), Retrosplenial Complex (RSC), and Occipital Place Area (OPA). Within the MNI-2 × 2 × 2 mm space, seed points were defined at the peak voxels within the intact > scrambled statistical map for each region (PPA, RSC, OPA) in each hemisphere. For a given seed, a flood fill algorithm was used to identify a cluster of spatially contiguous voxels around that seed which exceeded a given threshold. This threshold was then iteratively adjusted till a cluster size of approximately 500 voxels was achieved (corresponding to a volume of 4000 mm^3^); actual cluster sizes ranged from 499–502 voxels as an optimal solution to the algorithm was not always achievable. This step ensures that estimates of multi-voxel pattern similarity are not biased by the different sizes of ROIs being compared. Clusters were combined across hemispheres to yield 3 ROIs, each comprising approximately 1000 voxels. The locations of these peak voxels were similar to those which have been reported in previous fMRI studies^1–3^ – see also Supplementary Table S1. In addition, a V1 control ROI was defined from a recent standard atlas of retinotopic regions^51^.

Next, we measured patterns of response to the different stimulus conditions in the experiment. Parameter estimates were generated for each condition in the experimental scans. The reliability of response patterns was tested using a leave-one-participant-out (LOPO) cross-validation paradigm in which parameter estimates were determined using a group analysis of all participants except one. This generated parameter estimates for each scene condition in each voxel. This LOPO process was repeated such that every participant was left out of a group analysis once. These data were then submitted to correlation-based pattern analyses^21^ implemented using the PyMVPA toolbox^52^ (http://www.pymvpa.org/). Parameter estimates were normalised by subtracting the voxel-wise mean response across all experimental conditions per fold of the cross-validation^21^. For each iteration of the LOPO cross-validation, the normalized patterns of response to each stimulus condition were correlated between the group and the left-out participant. This allowed us to determine whether there are reliable patterns of response that are consistent across individual participants.

To further examine the effects of the normalisation process, we also performed our main analyses on the original un-normalised parameter estimates. Results of these analyses are shown in Supplementary Figure S5. The overall magnitude of the within- and between-cluster correlation values was increased, whilst the range was also compressed. The ability to discriminate the scene clusters (tested by contrasting the within- over the between-cluster correlations) remained similar to the analyses of the normalised parameter estimates. However, representational analyses revealed that none of the models tested (GIST, semantic, or perceptual) were able to predict the neural responses for any region. Failure to normalise the parameter estimates therefore impaired the ability to model the more nuanced relationships between the scene clusters.

### Local Semantic Concept Model

We adapted the local semantic concept model proposed by^23^ to determine the semantic similarity of the scenes. Objects within each of the scenes were manually segmented and labelled using the LabelMe toolbox^53^. Objects were then re-labelled by one of 22 core object labels; these comprised all 16 labels employed by^23^ (*sky*, *water*, *foliage*, *mountain*, *snow*, *rock*, *sand*, *animal*, *hill*, *fog*, *cloud*, *grass*, *dirt*, *manmade object*, *canyon*, and *road*), plus an additional 6 labels (*manmade structure*, *people*, *footpath*/*paved area*, *room interior*, *foodstuff*, and *vehicle*) necessary to fully describe the scenes within our stimulus set. Figure 4a illustrates this process for an example image. For each image a vector of 22 values was constructed where each value indicates the proportion of pixels within the image occupied by a given object label. Each vector was then normalised to have a magnitude of 1. Finally, a correlation based similarity matrix was constructed from these vectors using a leave-one-image-out cross-validation procedure.

### Perceptual Model

Participants completed a post-scan behavioural test, following a minimum delay of one week after the scan session in order to reduce bias by familiarity with the scenes. Written consent was obtained for all participants and the study was approved by the University of York Psychology Department Ethics Committee. Participants performed a card sorting task^24^. Each participant was provided with a set of printed cards from the original set of scene images (60 images; 6 per cluster). Subsets were counterbalanced across participants. Participants were required to sort the cards into 10 stacks according to their perceptual similarity so that cards within a particular stack were ones that they perceived to all be similar to one another. The task was designed to allow participants as much freedom as possible to sort the cards however they wished. The precise definition of perceptual similarity was left deliberately vague to encourage participants to form their own interpretation. Card stacks were allowed to vary in size, and participants were allowed unlimited time to complete the task. In order to prevent the paradigm becoming a memory task, participants were required to stack cards next to one another so that they could always be seen.

Following the test, the number of cards from each of the scene clusters was counted for each of the card stacks. For each scene cluster a vector of 10 values was constructed representing the counts for that cluster across each of the card stacks. The lower-triangle of a perceptual similarity matrix was constructed by taking the dot-product of the vectors between each unique pairing of clusters, such that the element at position (*i*,*j*) represents the dot product between the vectors of the *i* ^*th*^ and *j* ^*th*^ scene clusters respectively. Values thus represent the frequency of co-occurrence between pairs of scene clusters across card stacks.

### Statistical Analyses

A Fisher’s z-transform was applied to the correlation similarity matrices (GIST, MVPA, semantic) before further statistical analyses. In all cases, statistical tests were two-tailed and employed an alpha criterion of 0.050 for determining statistical significance. A Holm-Bonferroni correction for multiple comparisons was applied across the 3 scene regions (PPA, RSC, OPA).

We first tested the MVPA and semantic models for their ability to decode the scene clusters. For each iteration of the cross-validation, we calculated the average within cluster (on-diagonal) and between cluster (off-diagonal) values of the correlations matrix. These values were then entered into a paired-samples t-test. If scene clusters can be discriminated, then significantly greater within- than between-cluster correlations would be expected.

We also conducted a series of representational similarity analyses (RSAs)^22^. Correlation matrices (GIST, MVPA, semantic) were averaged across iterations of the cross-validation, while the perceptual dot product matrices were averaged across subjects. Representational similarity was assessed by correlating off-diagonal elements of the averaged similarity matrices between each of the models. If any model is able to predict any other, a significant correlation would be expected between the respective similarity matrices.

To test for effects outside our ROIs, we also performed a series of whole-brain searchlight analyses^28^. A spherical ROI (10 mm radius) was iterated over the whole-brain volume, and the MVPA repeated within each sphere. To test discrimination of the scene clusters, for a given sphere an average within- and between-cluster correlation value was calculated for each LOPO iteration. A paired-samples t-test was then used to test the within- over between-cluster correlation difference across LOPO iterations, and the *p*-value of the test assigned to the central voxel of the sphere. We also performed the simple representational similarity analyses using the searchlight approach, comparing the MVPA similarity matrices against the GIST, local semantic concept, and perceptual models. For a given sphere, the MVPA correlation matrices were averaged over LOPO iterations, and then the off-diagonal elements correlated with those from each of the models. In each case the *p*-value of the test was then assigned to the central voxel of the sphere. Statistical maps were visualised on the Freesurfer average cortical surface.

## Electronic supplementary material

Supplementary Information
