# Supplementary material for: A data driven approach to understanding the organization of high-level visual cortex
Source: Sci Rep. 2017 Jun 15;7:3596. doi: 10.1038/s41598-017-03974-5 (PMC5472563; doi:10.1038/s41598-017-03974-5)
Supplement: Supplementary file 1 — Supplementary Information [file 41598_2017_3974_MOESM1_ESM.pdf]

# A DATA DRIVEN APPROACH TO UNDERSTANDING THE ORGANIZATION OF HIGH-LEVEL VISUAL CORTEX – SUPPLEMENTARY INFORMATION

David M. Watson*, Timothy J. Andrews and Tom Hartley

* Corresponding author: david.watson@nottingham.ac.uk

**SUPPLEMENTARY TABLES**

**Supplementary Table S1.** Peak MNI mm co-ordinates (x, y, z) of PPA, RSC, and OPA regions reported in literature.

| | | LH | RH |
|---|---|---|---|
| PPA | Dilks *et al.* (2011) | -25, -45, -6 | 27, -45, -8 |
| | Epstein *et al.* (1999) | -29, -40, -7 | 23, -40, -7 |
| | Epstein *et al.* (2003) | -27, -51, -9 | 31, -48, -12 |
| | Epstein and Higgins (2007) | -19, -37, -8 | 20, -36, -6 |
| | Golomb and Kanwisher (2011) | -28, -52, -10 | 28, -51, -12 |
| | Henderson *et al.* (2011) | -19, -42, -2 | 23, -41, -3 |
| | Julian *et al.* (2012) | -20, -42, -12 | 22, -42, -12 |
| | Köhler *et al.* (2002) | -12, -42, -2 | 21, -35, -11 |
| | Mullally and Maguire (2011) | -27, -42, -12 | 33, -39, -12 |
| | O'Craven and Kanwisher (2000) | -28, -39, -3 | 31, -39, -6 |
| | Park *et al.* (2007) | -26, -42, -12 | 26, -42, -11 |
| | | | |
| RSC | Dilks *et al.* (2011) | -19, -57, 15 | 21, -56, 6 |
| | Epstein and Higgins (2007) | -10, -59, 8 | 13, -54, 9 |
| | Julian *et al.* (2012) | -10, -54, 12 | 16, -50, 6 |
| | Park *et al.* (2007) | -16, -55, 20 | 15, -51, 22 |
| | Schinazi and Epstein (2010) | -22, -50, 6 | 17, -53, 12 |
| | | | |
| OPA | Dilks *et al.* (2011) | -34, -78, 27 | 38, -75, 26 |
| | Epstein and Higgins (2007) | -33, -79, 31 | 32, -75, 34 |
| | Hasson *et al.* (2003) | -35, -81, 18 | 37, -79, 16 |
| | Julian *et al.* (2012) | -32, -76, 24 | 36, -80, 20 |
| | Levy *et al.* (2004) | -36, -80, 17 | 36, -78, 19 |

**Supplementary Table S2.** Results of representational similarity analyses using semi-partial correlations. Pearson's correlations were computed between the given outcome and predictor variables, holding the control variable constant for the predictor variable, but leaving the variance between the control and outcome variables unfiltered. These analyses are complimentary to the partial correlation analyses reported in the main text, and in all cases yielded similar results. (***$p < .001$, ** $p < .01$, * $p < .05$).

| Outcome | Predictor | Control | r | DOF | *p* | Sig |
|---|---|---|---|---|---|---|
| Perceptual | GIST | Semantic | .10 | 42 | .512 | ns |
| Perceptual | Semantic | GIST | .63 | 42 | < .001 | *** |
| | | | | | | |
| MVPA (PPA) | GIST | Perceptual | .33 | 42 | .031 | * |
| MVPA (PPA) | Perceptual | GIST | .30 | 42 | .044 | * |
| | | | | | | |
| MVPA (V1) | GIST | Semantic | .49 | 42 | < .001 | *** |
| MVPA (V1) | Semantic | GIST | .33 | 42 | .030 | * |
| | | | | | | |
| MVPA (V1) | GIST | Perceptual | .54 | 42 | < .001 | *** |
| MVPA (V1) | Perceptual | GIST | .14 | 42 | .348 | ns |

**SUPPLEMENTARY FIGURES**

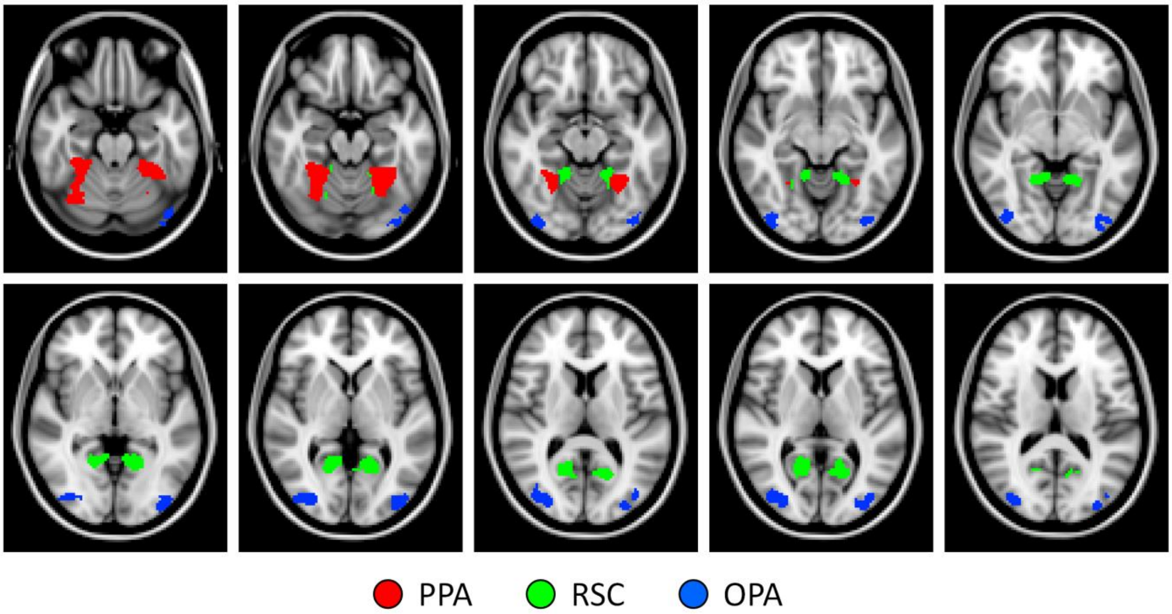


**Supplementary Figure S1.** Illustration of the masks used for the fMRI analyses. Each mask comprises approximately 500 voxels (4000mm$^3$) in each hemisphere. Slices of MNI brain span the range from z = -22mm to z = +18mm in 4mm increments.

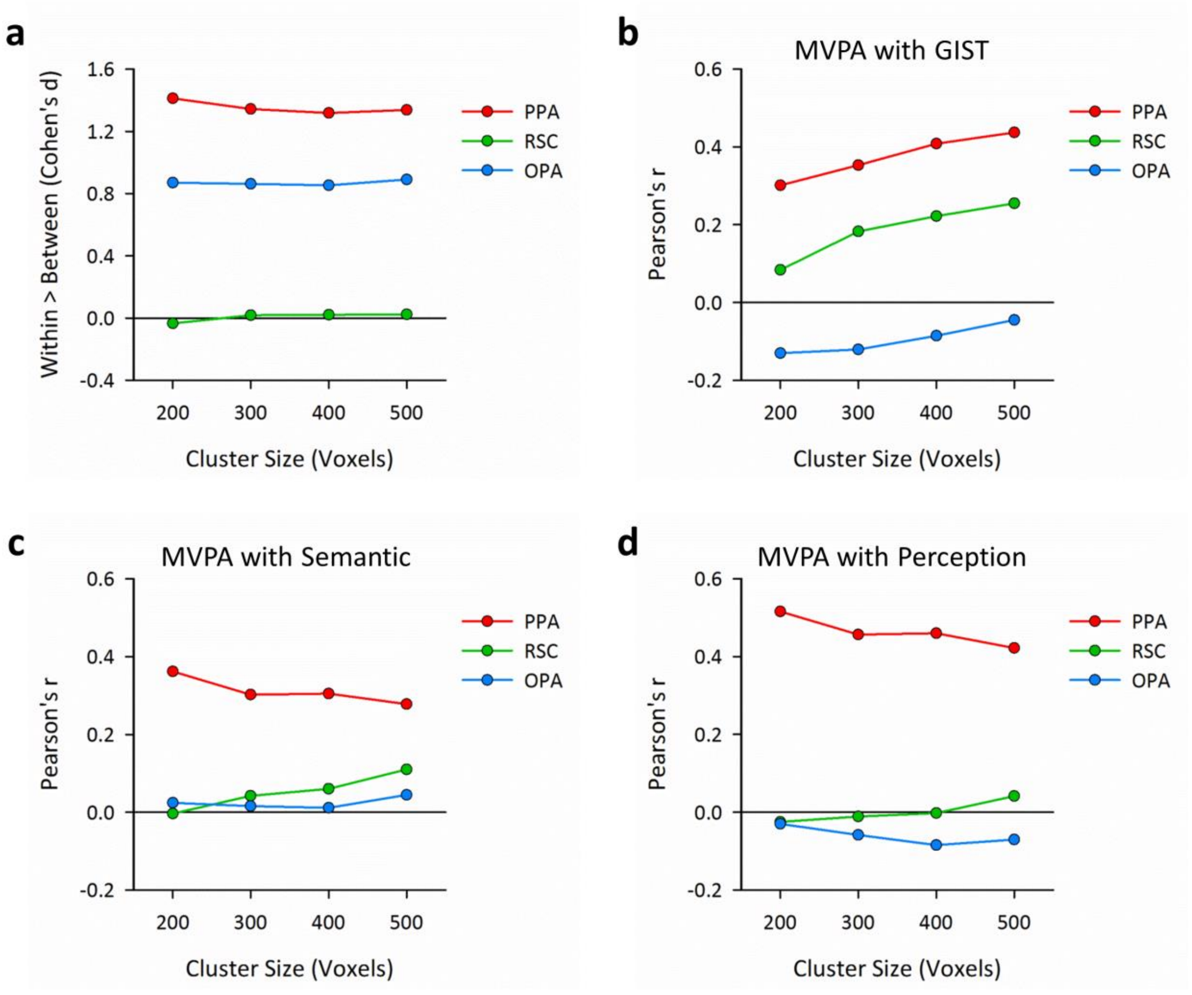


**Supplementary Figure S2.** Effects of ROI cluster size on analyses. For each region (PPA, RSC, OPA), clusters of spatially contiguous voxels were defined around seed points in each hemisphere. Clusters of 200, 300, 400, and 500 voxels were identified, which when combined across hemispheres yielded ROIs comprising 400, 600, 800, and 1000 voxels respectively. (a) Effect sizes for discrimination of scene clusters by contrasting within- over between-cluster correlation values. (b-d) Effect sizes for representational similarity analyses comparing the off-diagonal elements of the MVPA similarity matrices with the: (b) GIST, (c) semantic, and (d) perceptual models.

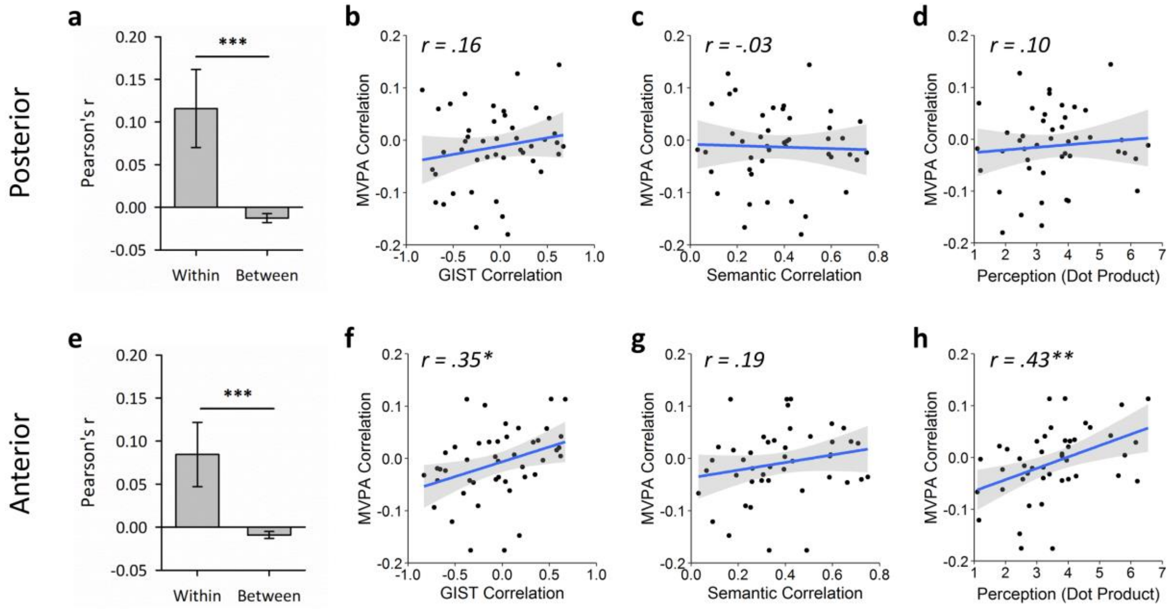


**Supplementary Figure S3.** Analysis of posterior (a-d) and anterior (e-h) sub-divisions of the PPA region. The PPA ROI was divided exactly halfway along its y-extent, and analyses repeated for each sub-division separately. A Holm-Bonferroni correction for multiple comparisons was applied across the two sub-divisions. (a, e) Average within and between cluster correlation values; error bars indicate 95% confidence intervals. Discrimination of clusters was assessed by paired-samples t-tests contrasting within- over between-cluster correlation values. Significantly greater within- than between-cluster correlations were observed in both the posterior (t(19) = 5.30, $p$ < .001, Cohen's d = 1.18) and anterior sub-divisions (t(19) = 4.74, $p$ < .001, Cohen's d = 1.06). Scatter plots show results of representational similarity analyses correlating off-diagonal elements of the MVPA similarity matrices against those of the (b, f) GIST, (c, g) semantic, and (d, h) perceptual models; shaded regions indicate 95% confidence intervals. In the anterior sub-division, there were significant correlations between neural responses and the GIST (r(43) = .35, $p$ = .036) and perceptual models (r(43) = .43, $p$ = .006), but not the semantic model (r(43) = .19, $p$ = .439). No models significantly predicted neural responses in the posterior sub-division (GIST: r(43) = .16, $p$ = .280; semantic: r(43) = -.03, $p$ = .832; perception: r(43) = .10, $p$ = .533).

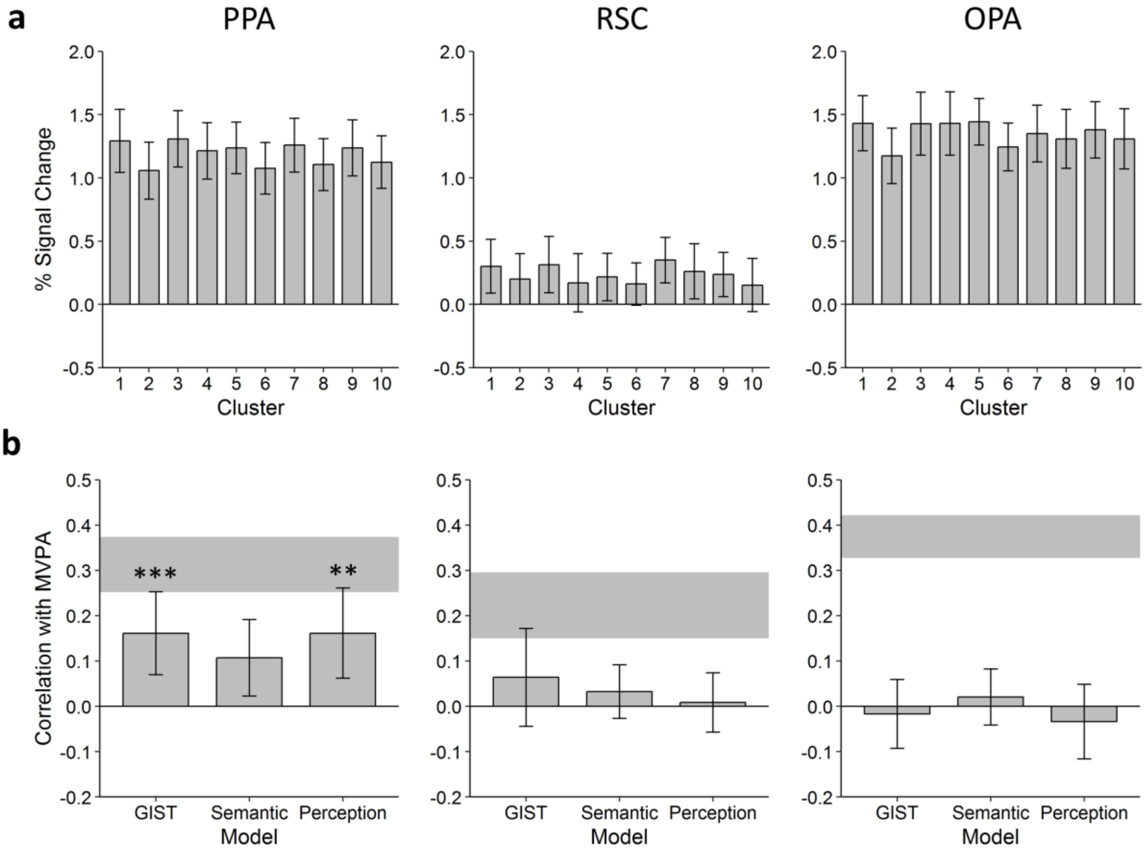


**Supplementary Figure S4.** Reliability of responses in the main scene ROIs. (a) Mean univariate amplitude of response in each ROI; error bars indicate 95% confidence intervals. Responses were significantly greater than zero ($p < .05$) for all scene clusters in PPA and OPA, and for all but clusters 4, 6, and 10 in RSC. (b) Reliability and variability of representational similarity analyses. For each ROI, each model was correlated against the MVPA similarity matrix for each LOPO iteration in turn, thereby yielding a distribution of 20 r-values per comparison. Bars indicate means of those distributions; error bars indicate 95% confidence intervals. Distributions were contrasted against zero using one-sample t-tests and Holm-Bonferroni corrected for multiple comparisons across ROIs. In the PPA, correlations were significantly greater than zero for the GIST ($t(19) = 3.62$, $p = .005$, Cohen's d = 0.81) and perceptual models ($t(19) = 3.30$, $p = .011$, Cohen's d = 0.74), whilst the semantic model approached significance ($t(19) = 2.61$, $p = .052$, Cohen's d = 0.58). No other comparisons were significant (all $p > .05$). Shaded regions indicate noise ceilings for each ROI, which provide estimates of the maximum possible correlation with the MVPA similarity matrices that is achievable given the inherent noise in the data. The upper bound of the noise ceiling is defined as the average correlation between each MVPA similarity matrix (across LOPO iterations) with the group average similarity matrix, whilst the lower bound is defined as the average correlation between each similarity matrix and the group average of all matrices excluding that one.

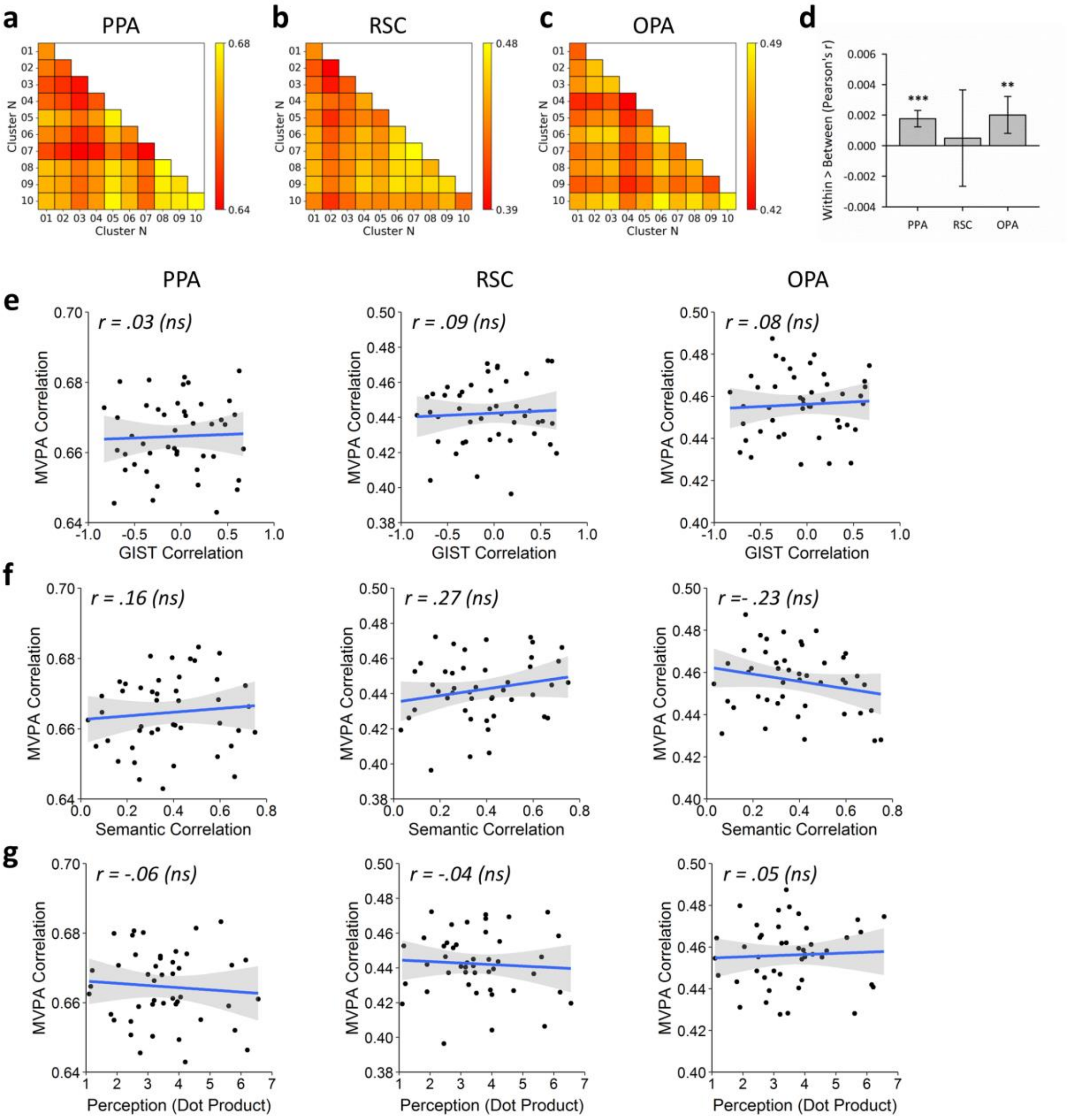


**Supplementary Figure S5.** MVPA of un-normalised parameter estimates. (a-c) Correlation matrices for each of the scene ROIs; note that the overall magnitude of correlations is increased and the range is compressed (cf. Figure 3a). (b) Discrimination of scene clusters by contrasting within- over between-cluster correlations; error bars show 95% confidence intervals. Although the magnitude of correlation differences is small, the variance has also been compressed, and so results still replicate those of the main analyses. Significantly greater within- than between-cluster correlations were observed in the PPA ($t(19) = 4.60$, $p < .001$, Cohen's d = 1.03) and OPA ($t(19) = 3.33$, $p = .007$, Cohen's d = 0.74), but not RSC ($t(19) = 0.15$, $p = .880$, Cohen's d = 0.03). (e-f) Scatter plots show representational similarity analyses comparing MVPA similarity matrices against the (e) GIST, (f) semantic, and (g) perceptual models; shaded regions indicate 95% confidence intervals. No comparisons were significant for any region (all $p > .05$). Failure to normalise the data therefore impaired the ability to model the more nuanced relationships between the scene clusters.
